# Basophils as a potential marker of lupus nephritis by flow cytometry

**DOI:** 10.2144/fsoa-2020-0212

**Published:** 2021-02-16

**Authors:** Yanni Jiang, Jin Chen, Yi Zhao, Yi Liu, Hong Xu, Xianming Mo

**Affiliations:** 1 Department of Rheumatology & Immunology, West China Hospital, Sichuan University, Chengdu, China; 2 Laboratory of Stem Cell Biology, State Key Laboratory of Biotherapy, West China Hospital, Sichuan University & Collaborative Innovation Center for Biotherapy, Chengdu, China

**Keywords:** basophils, CD4^−^CD8^−^ T cells, immunophenotype, innate lymphoid cells, lupus nephritis, systemic lupus erythematosus

## Abstract

**Objective:**

To establish a convenient and simple flow cytometry immunophenotyping panel to explore immune cellular alterations and potential cellular biomarkers in systemic lupus erythematosus.

**Materials and methods:**

This is a cross-sectional, case–control study including 60 patients with systemic lupus erythematosus and 20 sex- and age-matched healthy controls. A 14-color immunophenotyping panel was applied to detect proportions of circulating immune mononuclear cells, and comparisons between patients and healthy controls, and subgroups of patients, were performed. Correlations between cellular proportions and other parameters were investigated.

**Results:**

After multivariate analysis, significantly decreased proportions of CD4^−^CD8^−^ T cells, natural killer cells and innate lymphoid cells were observed in patients compared with healthy controls. The proportions of basophils were decreased significantly in patients with lupus nephritis (LN) compared with those in patients without LN.

**Conclusion:**

In the present study, we found that basophil proportions may be a biomarker of LN.

## Lay abstract

Systemic lupus erythematosus is a chronic, multisystem, autoimmune disorder that involves various abnormalities of immune cells and thus presents in a striking variety of ways. This study aimed to establish a biomarker panel that would enable the exploration of changes in immune cells and the relationships between immune cell subsets and clinical manifestations in patients with systemic lupus erythematosus. Our results showed that basophil cell proportions may be a biomarker of use in lupus nephritis.

Systemic lupus erythematosus (SLE) is a chronic, multisystem, autoimmune disorder characterized by overproduction of autoantibodies and type I interferons; it typically affects women between puberty and menopause [1]. Abnormalities in immune cells can occur in multiple parts of the immune response, resulting in striking heterogeneity in clinical presentation [2].

Studies reported that increased self-reactive B cells in patients with SLE directly cause overproduction of autoantibodies [3]. T cells participate in the development of lupus by promoting the secretion of proinflammatory cytokines and activating B cells to produce autoantibodies [4]. Furthermore, increased spontaneous apoptosis of T cells has been observed in patients with SLE [4]. An increased proportion of activated CD8^+^ T lymphocytes has also been reported in patients with active SLE [5]. Moreover, previous studies have suggested that CD4^−^CD8^−^ T cells are associated with lupus nephritis (LN) and that CD4^+^CD8^+^ T cells were associated with the Systemic Lupus International Collaborating Clinics damage index [6,7]. Regulatory T cells (Tregs) induce immunological tolerance and inhibit the pathogenesis of SLE [4].

In addition to the adaptive cells mentioned above, diverse types of innate immune cells participate in lupus development. Cumulative evidence shows that neutrophils – especially low-density granulocytes – take part in the pathogenesis of SLE through neutrophil extracellular trap formation, phagocytosis and response to type I interferon stimulation [8,9]. Basophils expressing the IgE receptor, FcεRI, are positively correlated with IgE autoreactivity in patients with active SLE [10]. Dendritic cells (DCs), particularly plasmacytoid dendritic cells (pDCs) which produce type I interferon, are also involved in the pathogenesis of SLE [11]. Recently, one study reported that the more severe forms of LN were correlated with lower levels of peripheral nonclassical monocytes [11]. Furthermore, natural killer (NK) cells and innate lymphoid cells (ILCs), belonging to the innate immune system, are important in the regulation of barrier homeostasis [12]. NK cells secrete cytokines, predominantly type II interferon, and their numbers have been reported to be decreased in patients with SLE [13]. To date, two studies have detected altered ILC proportions in patients with SLE [14,15]. Taken together, these findings suggest that SLE is a heterogeneous autoimmune disease.

SLE is a chronic disease treated with long-term steroids and/or immunosuppressants; thus it is necessary to detect and monitor patients’ immune cellular compositions in clinical practice. In the present study we first aimed to establish a feasible 14-color flow cytometry panel for clinical practice to detect multiple immune cellular compositions in peripheral mononuclear cells. Second, we explored alterations in immune cells between patients with SLE and healthy controls, and associations between immune cell type proportions and clinical features.

## Materials & methods

### Patients

We conducted a cross-sectional, case–control study. Sixty patients with SLE from the Department of Rheumatology and Immunology at West China Hospital of Sichuan University who fulfilled the 1997 American College of Rheumatology classification criteria for SLE [16] were enrolled in this study between January 2017 and April 2018. We also recruited 20 sex- and age-matched healthy donors.

Disease activity was measured using the SLE Disease Activity Index 2000 (SLEDAI-2000), and a score ≥5 was defined as active [17]. Organ system involvement was identified according to 1997 American College of Rheumatology criteria. The laboratory tests included anti-nuclear antibody titer, anti-dsDNA antibody titer, anti-Sjögren syndrome-related antigen A (SSA) antibody, anti-Sjögren syndrome-related antigen B (SSB) antibody, anti-RNP antibody, anti-Smith (Sm) antibody, Coombs test, IgG, IgM, IgA, IgE, serum complement component 3 (C3), serum complement component 4 (C4), serum CRP and erythrocyte sedimentation rate. Disease manifestations and ongoing therapy were recorded in the inclusion visit.

### Flow cytometric analysis

Anticoagulated peripheral blood samples (1 ml) were collected into EDTA-2K from patients with SLE and healthy controls. The red blood cells were removed by osmotic lysis of erythrocytes with pH 7 phosphate-buffered saline to obtain peripheral white blood cells. Human Trustain FcX™ (BioLegend, CA, USA) was used to block the Fc receptor for 5 min prior to antibody staining. To mitigate nonspecific interactions between multiple BD Horizon Brilliant™ polymer conjugates, BD Horizon Brilliant Stain Buffer (BD Biosciences, NJ, USA) was used according to the manufacturer’s instructions. After adding all reagents, the cell suspension was incubated at room temperature in the dark for 15 min and measured with a BD FACSAia SORP flow cytometer (BD Biosciences) after washing twice with phosphate-buffered saline. The cytometry data were analyzed with FlowJo X software (TreeStar, OR, USA). Gating was based on isotype controls and fluorescence minus one control. The total events of each sample were >500,000. For adjusting compensation, we utilized the automatic adjusting compensation model of the machine BD FACSAia SORP, and then we applied artificial compensation based on each cell subset. The detailed compensation matrix is shown in Supplementary Figure 1.

### Statistical analysis

All statistical analyses were performed using SPSS v. 19.0 software (SPSS Inc., IL, USA). Continuous variables were expressed as the mean ± standard deviation; categorical variables were reported as a percentage (%). Differences between two groups were compared by Student *t *test for normally distributed data, Mann–Whitney U-test for nonparametric distribution data and Fisher exact test for categorical variables. Differences among three study groups were analyzed by Kruskal–Wallis test followed by Dunn’s test. Correlation analysis was evaluated using the Pearson correlation coefficient for normally distributed data and Spearman correlation coefficient for abnormally distributed data. Logistic or linear regression analysis was performed using positive variables by univariate analysis. A p-value < 0.05 was considered statistically significant.

## Results

### Development of a 14-color flow cytometry immunophenotyping panel

**Figure 1.**
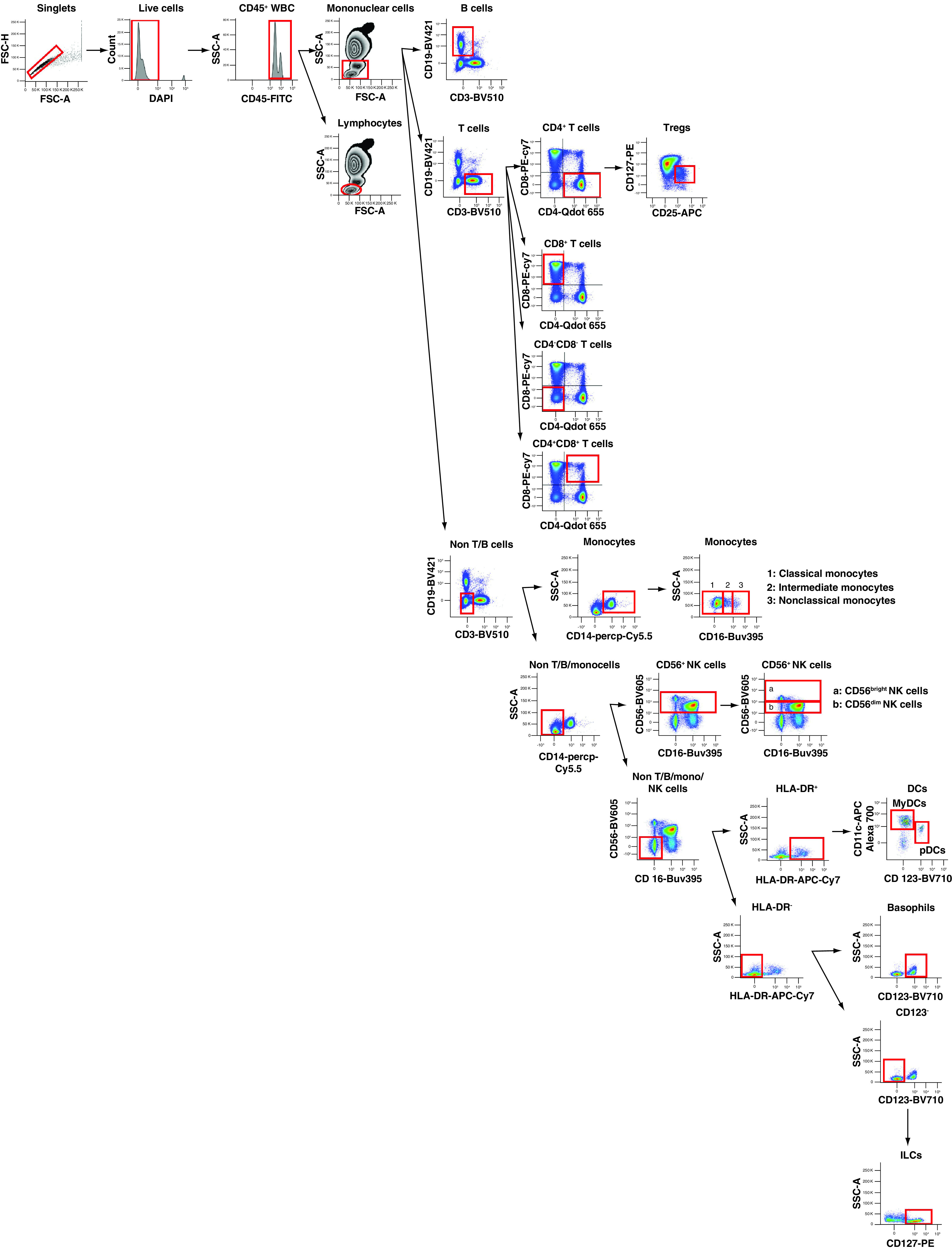
Flow cytometry panel from human peripheral blood. After obtaining the sample, the red cells underwent lysis. The 14-color panel was performed on whole blood cells. Subpopulations of peripheral immune mononuclear cells (T cells, CD4^+^ T cells, CD8^+^ T cells, CD4^−^CD8^−^ T cells, CD4^+^CD8^+^ T cells, Tregs, B cells, NK cells, ILCs, basophils, MyDCs, pDCs, monocytes, classical [CD14^+^CD16^−^], intermediate [CD14^+^CD16^low^] and nonclassical [CD14^+^CD16^hi^] monocytes) were identified. The cellular gating depicted is representative of one healthy individual sample. DC: Dendritic cell; FSC-A: Forward scatter area; FSC-H: Forward scatter height; HLA-DR: Human leukocyte antigen DR; ILC: Innate lymphoid cell; MyDC: Myeloid dendritic cell; NK: Natural killer; pDC: Plasmacytoid dendritic cell; SSC: Side scatter; Treg: Regulatory T cell; WBC: White blood cell.

Although there have been approaches covering nearly 30 surface biomarkers to identify circulating or urine phenotype of patients with SLE [18], we aimed to establish a feasible multiparametric flow cytometry panel in Chinese real-life clinical practice. Therefore we established a 14-color panel to detect peripheral basophils, monocytes, classical/intermediate/nonclassical monocytes, pDCs, myeloid dendritic cells (MyDCs), B cells, T cells, CD4^+^ T cells, CD8^+^ T cells, CD4^+^CD8^+^ T cells, CD4^−^CD8^−^ T cells, NK cells and ILCs. The 14-color immunophenotyping panel and gating strategy are shown in Table 1 & Figure 1, respectively. Figure 1 shows that live leukocytes were identified first based on the expression of CD45; we used DAPI to exclude dead cells. Mononuclear cells were differentiated from granulocytes based on forward (size) and side (granularity) scatter characteristics. B cells were identified as mononuclear cells expressing CD19 and lacking CD3. T cells were gated as mononuclear cells that did not express CD19 but expressed CD3; these cells were subdivided based on CD4 and CD8 into CD4^+^ T cells, CD8^+^ T cells, CD4^−^CD8^−^ T cells and CD4^+^CD8^+^T cells. Tregs were gated as CD4^+^ T cells expressing high levels of CD25 and low levels of CD127 (CD25^hi^CD127^low^CD4^+^ T cells). Monocytes were gated as mononuclear cells that did not express CD3 and CD19 but expressed CD14; these cells were subdivided based on the CD16 mean fluorescent intensity histogram into classical (CD14^+^CD16^−^), intermediate (CD14^+^CD16^low^) and nonclassical (CD14^+^CD16^hi^) monocytes [19,20]. NK cells were identified as mononuclear cells that did not express CD3, CD19 or CD14 but expressed CD56, and were further subdivided into CD56^dim^ and CD56^bright^ NK cells depending on the CD56 MFI histogram [21]. DCs were identified as mononuclear cells expressing the human leukocyte antigen-DR isotype (HLA-DR) but lacking lineage markers (CD3, CD14, CD19, CD16 and CD56). DCs were further subdivided into myeloid DCs (expressing CD11c) and plasmacytoid DCs (expressing CD123). Cells lacking lineage markers and HLA-DR but expressing CD123 were gated as basophils. ILCs were identified as mononuclear cells lacking the lineage markers CD11c, CD123 and HLA-DR, but expressing CD127 [22]. The definitions of all immune cells are shown in Supplementary Table 1.

**Table 1.** Optimized combination of 14 commercially available fluorochrome-conjugated antibodies to reliably quantify 18 subpopulations of immune cells.

Conjugation	Antibody	Clone (company)	Titer
FITC	Anti-human CD45 antibody	HI30 (BioLegend)	1:50
Percp-cy5.5	Anti-human CD14 antibody	M5E2 (BD)	1:50
BV510	Anti-human CD3 antibody	UCHT1 (BD)	1:50
Qdot655	Anti-human CD4 antibody	S3.5 (Invitrogen)	1:200
PE-CY7	Anti-human CD8 antibody	RPA-T8 (BD)	1:100
APC	Anti-human CD25 antibody	BC96 (BioLegend)	1:20
BUV395	Anti-human CD16 antibody	3G8 (BD)	1:100
BV605	Anti-human CD56 antibody	NCAM16.2	1:100
APC-CY7	Anti-human HLA-DR antibody	L243 (BioLegend)	1:100
BV421	Anti-human CD19 antibody	HIB19 (BD)	1:100
PE	Anti-human CD127 antibody	A019D5 (BioLegend)	1:25
BV710	Anti-human CD123 antibody	9F5 (BD)	1:50
Alexa Fluor^®^ 700	Anti-human CD11c antibody	Bu15 (BioLegend)	1:50
DAPI	Live/dead		1:50

### Characteristics of patients with SLE

Next we conducted immunophenotyping of peripheral mononuclear cells in 60 patients with SLE and 20 healthy controls. All patients were Han Chinese with a mean age of 35.27 years and 81.67% (49) were female, with a mean SLEDAI-2000 of 6.2 and mean disease duration of 5.09 years. Thirty-two patients had active SLE and 28 patients had inactive SLE. Fifty-eight patients were administered with glucocorticoid with an equivalent daily prednisone dose of 33.67 ± 25.53 mg/day. Twenty patients were administered with cyclophosphamide with a cumulative dose of 2.25 ± 2.16 g. The leukocyte and granulocyte counts were 7.33 ± 0.61 × 10^9^/l and 5.50 ± 0.54 × 10^9^/l, respectively. The demographic, clinical and serological features of the subjects are summarized in Table 2.

**Table 2.** Demographic, clinical and serological features of patients with systemic lupus erythematosus.

Characteristic	SLE (n = 60)	HC (n = 20)	p-value
Age (years)	35.27 ± 12.21	30.90 ± 8.58	0.09
Sex, n (% female)	49 (81.67)	15 (75)	0.53
Disease duration (years)	5.09 ± 0.92		
Current concomitant medications	58 (96.67)		
Hydroxychloroquine, n (%)	36 (60)		
Prednisone, n (%)	58 (96.67)		
Current dose of prednisone (mg/day)	33.67 ± 25.53		
Cyclophosphamide, n (%)	20 (33.33)		
Cumulate dose of cyclophosphamide (g)	2.25 ± 2.16		
Mycophenolate mofetil	3 (5)		
Leflunomide	1 (1.67)		
SLEDAI-2000	6.2 ± 4.46		
Active SLE patients (SLEDAI-2000 ≥5)	32 (53.33)		
Mean SLEDAI-2000 scores	9.53 ± 3.25		
Inactive SLE patients (SLEDAI-2000 <5)	28 (46.67)		
Mean SLEDAI-2000 scores	2.39 ± 1.77		
System involved, n (% positive)			
Mucocutaneous involvement	26 (43.33)		
Arthritis	21 (35)		
Nephritis	38 (63.33)		
Hematological involvement	25 (41.67)		
Laboratory features, n (% positive)			
ANA	60 (100)		
ANA titer <1:1000	27 (45)		
ANA titer ≥1:1000	33 (55)		
Anti-dsDNA antibody	34 (56.67)		
Anti-dsDNA titer <1:100	21 (35)		
Anti-dsDNA titer ≥1:100	13 (21.67)		
Anti-SSA antibody	35 (58.33)		
Anti-SSB antibody	21 (35)		
Anti-Sm antibody	29 (48.33)		
Anti-RNP antibody	35 (58.33)		
C3 (g/l)	0.56 ± 0.04		
C4 (g/l)	0.12 ± 0.01		
IgG (g/l)	15.17 ± 9.41		
IgA (mg/l)	2597.08 ± 1438.96		
IgM (mg/l)	1134.98 ± 939.55		
IgE (IU/ml)	47.06 ± 64.7		
ESR (mm/h)	33.78 ± 20.8		
CRP (mg/dl)	8.67 ± 15.45		
Leukocyte count (10^9^/l)	7.33 ± 0.61		
Lymphocyte count (10^9^/l)	1.29 ± 0.26		
Granulocyte count (10^9^/l)	5.50 ± 0.54		

Data are expressed as absolute numbers (%) or the mean ± standard deviation.

ANA: Anti-nuclear antibody; C3/4: Complement component 3/4; ESR: Erythrocyte sedimentation rate; HC: Healthy control; SLE: Systemic lupus erythematosus; SLEDAI-2000: SLE Disease Activity Index 2000; Sm: Smith; SSA: Sjögren syndrome-related antigen A; SSB: Sjögren syndrome-related antigen B.

### Differences in immune cellular compositions between patients with SLE & healthy controls

The proportions of CD4^+^ T cells, CD8^+^ T cells, CD4^−^CD8^−^ T cells, CD4^+^CD8^+^ T cells, Tregs, NK cells, MyDCs, pDCs, basophils and ILCs were altered in patients with SLE compared with those in healthy controls by univariate analysis. However, after multivariate analysis, only the proportions of CD4^−^CD8^−^ T cells, NK cells and ILCs were significantly decreased in patients with SLE compared with those in healthy controls (p = 0.027, p < 0.001 and p = 0.014, respectively) (Table 3).

**Table 3.** Differences in immune cellular compositions between patients with systemic lupus erythematosus and healthy controls.

Variables	SLE (n = 60)	HC (n = 20)	p-value	
			Univariate	Multivariate
B cells	17.41 ± 11.1	12.56 ± 4.02	NS	
T cells	76.17 ± 11.87	73.96 ± 8.32	NS	
CD4^+^ T cells	43.59 ± 13.93	54.00 ± 8.18	<0.001	0.22
CD8^+^ T cells	49.81 ± 14.53	36.61 ± 8.12	<0.001	0.22
CD4^−^CD8^−^ T cells	5.12 ± 3.67	8.61 ± 4.94	<0.001	0.027
CD4^+^CD8^+^ T cells	1.47 ± 1.54	0.78 ± 0.34	0.028	0.379
Tregs	4.31 ± 2.00	6.18 ± 1.31	<0.001	0.095
Monocytes	1.48 ± 1.56	1.58 ± 1.14	NS	
Classical monocytes (CD14^+^CD16^−^)	76.24 ± 14.84	80.33 ± 14.62	NS	
Intermediate monocytes (CD14^+^CD16^low^)	11.41 ± 7.10	12.65 ± 9.25	NS	
Nonclassical monocytes (CD14^+^CD16^hi^)	11.54 ± 12.16	6.64 ± 5.80	NS	
NK cells	4.70 ± 3.97	11.85 ± 7.46	<0.001	<0.001
CD56^dim^ NK cells	85.18 ± 12.14	91.23 ± 6.46	NS	
CD56^bright^ NK cells	14.82 ± 12.14	8.77 ± 6.46	NS	
MyDCs	0.05 ± 0.05	0.10 ± 0.05	<0.001	0.571
pDCs	0.01 ± 0.01	0.05 ± 0.03	<0.001	0.092
Basophils	0.03 ± 0.09	0.12 ± 0.12	<0.001	0.649
ILCs	0.47 ± 0.38	0.94 ± 0.60	<0.001	0.014

Data of monocytes, MyDCs, pDCs and basophils are expressed as a percentage of live CD45^+^ white blood cells. Data for B cells, T cells, NK cells and ILCs are expressed as a percentage of live lymphocytes. Data are shown as the mean ± standard deviation. p < 0.05 indicates statistical significance.

HC: Healthy control; ILC: Innate lymphoid cell; MyDC: Myeloid dendritic cell; NK: Natural killer; NS: Not significant; pDC: Plasmacytoid dendritic cell; SLE: Systemic lupus erythematosus.

### Associations between immune cellular composition & SLE disease activity

In the present study, 32 patients had active SLE and 28 had inactive SLE. Demographic, clinical and serological data of the two groups are summarized in Supplementary Table 2. The age and SLEDAI-2000 score differed significantly between the two groups. In univariate analysis, altered proportions of CD4^+^ T cells and CD8^+^ T cells were found between patients with active SLE and those with inactive SLE; the proportions of CD4^+^ T cells, CD8^+^ T cells, CD4^−^CD8^−^ T cells, CD4^+^CD8^+^ T cells, Tregs, NK cells, MyDCs, pDCs, basophils and ILCs were altered in patients with active SLE compared with those in healthy controls; and the proportions of Tregs, NK cells, MyDCs, pDCs, basophils and ILCs were altered in patients with inactive SLE compared with those in healthy controls. However, after multivariate analysis, there were no differences in immune cellular compositions among these three groups (Table 4). Furthermore, no immune cell populations were correlated with the SLEDAI-2000 score (data not shown).

**Table 4.** Differences in immune cellular compositions among patients with active systemic lupus erythematosus, patients with inactive systemic lupus erythematosus and healthy controls.

Variables	Active SLE (n = 32)	Inactive SLE (n = 28)	HC (n = 20)	p-value^†^		p-value^‡^		p-value^§^	
				Univariate	Multivariate	Univariate	Multivariate	Univariate	Multivariate
B cells	17.37 ± 12.13	17.45 ± 10.01	12.56 ± 4.02	NS		NS		NS	
T cells	76.37 ± 12.97	75.95 ± 10.7	73.96 ± 8.32	NS		NS		NS	
CD4^+^ T cells	38.36 ± 9.67	49.57 ± 15.72	54.00 ± 8.18	<0.01	0.65	<0.001	0.64	NS	
CD8^+^ T cells	55.69 ± 10.88	43.09 ± 15.42	36.61 ± 8.12	<0.01	0.19	<0.001	0.64	NS	
CD4^−^CD8^−^ T cells	4.2 ± 2.34	6.18 ± 4.58	8.61 ± 4.94	NS		<0.001	0.64	NS	
CD4^+^CD8^+^ T cells	1.75 ± 1.9	1.16 ± 0.9	0.78 ± 0.34	NS		<0.05	0.66	NS	
Tregs	4.62 ± 2.1	3.95 ± 1.86	6.18 ± 1.31	NS		<0.001	0.85	<0.001	0.15
Monocytes	1.85 ± 1.76	1.06 ± 1.19	1.58 ± 1.14	NS		NS		NS	
Classical monocytes (CD14^+^CD16^−^)	76.28 ± 16.2	76.19 ± 13.42	80.33 ± 14.62	NS		NS		NS	
Intermediate monocytes (CD14^+^CD16^low^)	10.38 ± 6.69	12.6 ± 7.48	12.65 ± 9.25	NS		NS		NS	
Nonclassical monocytes (CD14^+^CD16^hi^)	12.69 ± 13.72	10.22 ± 10.17	6.64 ± 5.80	NS		NS		NS	
NK	4.79 ± 4.38	4.6 ± 3.51	11.85 ± 7.46	NS		<0.001	0.15	<0.001	0.07
CD56^dim^ NK cells	86.1 ± 13.22	84.13 ± 10.91	91.23 ± 6.46	NS		NS		NS	
CD56^bright^ NK cells	13.9 ± 13.22	15.87 ± 10.91	8.77 ± 6.46	NS		NS		NS	
MyDCs	0.05 ± 0.06	0.04 ± 0.04	0.10 ± 0.05	NS		<0.001	0.51	<0.001	0.16
pDCs	0.01 ± 0.01	0.02 ± 0.02	0.05 ± 0.03	NS		<0.001	0.53	<0.001	0.83
Basophils	0.03 ± 0.06	0.04 ± 0.12	0.12 ± 0.12	NS		<0.001	0.81	<0.01	0.54
CD127^+^ ILCs	0.43 ± 0.33	0.51 ± 0.44	0.94 ± 0.60	NS		<0.001	0.79	<0.01	0.23

Data for monocytes, MyDCs, pDCs and basophils are expressed as a percentage of live CD45^+^ white blood cells. Data for B cells, T cells, NK cells and ILCs are expressed as a percentage of live lymphocytes. Data for CD4^+^ T cells, CD8^+^ T cells, CD4^+^CD8^+^ T cells, CD4^−^CD8^−^ T cells, Tregs, classical/intermediate/nonclassical monocytes, CD56^dim^ NK cells and CD56^bright^ NK cells are expressed as a percentage of the parent cells. Data are shown as the mean ± standard deviation. p < 0.05 indicates statistical significance.

† active SLE versus inactive SLE.

‡ active SLE versus HC.

§ inactive SLE versus HC.

HC: Healthy control; ILC: Innate lymphoid cell; MyDC: Myeloid dendritic cell; NK: Natural killer; NS: Not significant; pDC: Plasmacytoid dendritic cell; SLE: Systemic lupus erythematosus.

### Associations between immune cellular compositions & clinical & serological features of patients with SLE

**Figure 2.**
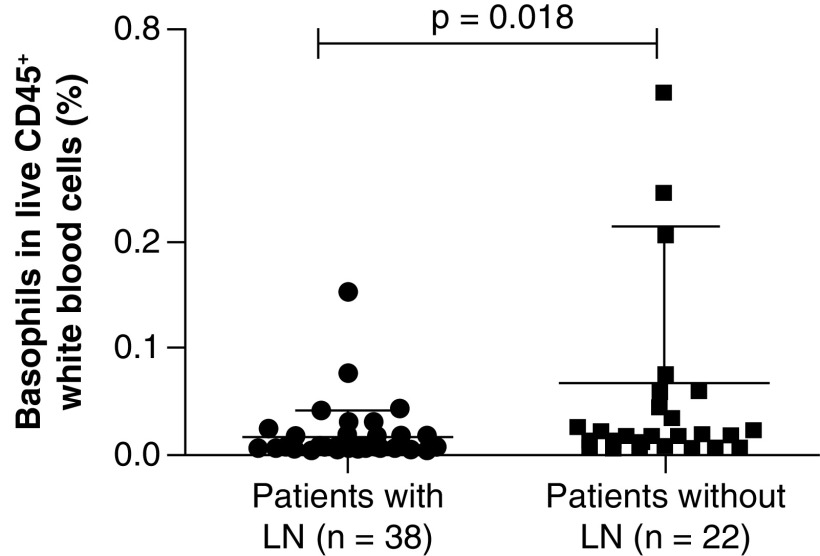
Differences in basophils between patients with and without lupus nephritis. The proportion of basophils was decreased in patients with nephritis (n = 38) compared with patients without nephritis (n = 22). Data are shown as the mean ± standard deviation. p < 0.05 indicates statistical significance.

We compared the proportions of all immune cell populations between patients with or without specific clinical (mucocutaneous involvements, arthritis, nephritis and hematological involvement), serological features (anti-nuclear antibody titer, anti-dsDNA antibody titer, anti-SSA antibody, anti-SSB antibody, anti-RNP antibody, anti-Smith antibody, serum C3 level, serum C4 level, IgG/IgM/IgA/IgE level) or inflammatory markers (erythrocyte sedimentation rate and CRP level). Demographic, clinical and serological data of patients with and without nephritis are summarized in Supplementary Table 3. Logistic regression analysis of positive variables in univariate analysis showed that basophil proportions were significantly lower in patients with nephritis than in patients without nephritis (p = 0.018; Figure 2). The proportions of all immune cells in the present study showed no correlation with serological features or inflammation markers of patients with SLE (data not shown).

## Discussion

SLE is a heterogeneous systemic disease characterized by a wide spectrum of serological features, clinical manifestations and degrees of severity. A landmark report conducted to evaluate the blood transcriptomes of 158 patients with SLE indicated that patients were divided into seven groups based on their genotypes, and revealed the molecular heterogeneity of SLE at the transcriptome level [23]. Another study used six eight-color panels with flow cytometry to assess the cellular heterogeneity of active SLE and found that active SLE patients could be divided into three subgroups based on T-cell heterogeneity [24]. It is clear that SLE is a complicated disease with clinical, transcriptional and cellular heterogeneity, and not all patients share the same abnormalities [25]. Thus it is difficult to develop effective targeted therapy for SLE. In the present study, to rapidly and conveniently conduct comprehensive immunophenotyping of peripheral mononuclear cells, we established a single tube test for 14-color flow cytometry to measure T cells, CD4^+^ T cells, CD8^+^ T cells, CD4^−^CD8^−^ T cells, CD4^+^CD8^+^ T cells, Tregs, B cells, NK cells, ILCs, basophils, MyDCs, pDCs, monocytes and classical/intermediate/nonclassical monocytes.

CD4^−^CD8^−^ T cells are defined as CD3^+^CD4^−^CD8^−^ T cells, which are Tregs that can downregulate immune responses and are composed of three subsets: natural killer T (NKT) cells (TCRαβ^+^CD1d^+^CD4^−^CD8^−^), γδ T cells (TCRγδ^+^) and double-negative T cells (TCRαβ^+^CD4^−^CD8^−^) [26]. A previous study revealed that expanded double-negative T cells infiltrated the kidney in patients with LN and produced inflammatory cytokine IL-17 [6]. The results showed that NKT cells were deficient in patients with SLE compared with healthy controls, and regulated the balance between Th1 and Th2 cells in SLE [27]. γδ T cells, accounting for 5–10% of total T cells, can secrete various types of cytokines (e.g., IFN-γ, IL-10 and IL-17) and can help B cells to produce antibodies [28]. The numbers of γδ T cells were decreased in the peripheral blood of patients with SLE compared with those in healthy controls [28]. These results demonstrate that CD4^−^CD8^−^ T-cell subsets are diverse and play different roles in SLE. In the present study we found that total CD4^−^CD8^−^ T-cell proportions were decreased in patients with SLE, but additional experiments are necessary to explore the immunophenotyping and alterations of CD4^−^CD8^−^ T cells in SLE.

ILCs are classified into two main groups: cytotoxic NK cells (CD127^−^) and noncytotoxic ILCs (CD127^+^) [12]. NK cells play a crucial role in infectious diseases and tumors by producing the immunoregulatory cytokine IFN-γ [12]. Human NK cells can be divided into two subsets (CD56^dim^ NK cells and CD56^bright^ NK cells) based on their relative expression of the adhesion molecule CD56 [21]. CD56^dim^ NK cells are the major type of human NK cells. CD56^dim^ NK cells and CD56^bright^ NK cells have different functions in cytotoxicity, cytokine production and proliferation [21], and it remains unclear whether NK cells play a pathogenetic role in SLE. Previous studies have shown that impaired cytotoxic function of NK cells and a decreased proportion of NK cells can be identified in patients with SLE [13]. Consistent with these studies, we observed a decreased proportion of NK cells in patients in SLE. Further studies are needed to explore how decreased NK cells are involved in the pathogenesis of SLE.

Two previous studies have observed increased proportions of ILCs in patients with SLE compared with proportions in healthy controls, and in patients with active SLE compared with patients with inactive SLE [14,15]. In contrast, the present study showed a decreased ILC proportion in patients with SLE. Additional studies in larger and different cohorts are necessary to validate these opposing findings.

LN is one of the most frequent severe forms of organ involvement in SLE; it is a major cause of end-stage renal disease and causes increased mortality and morbidity in patients with SLE [29]. Traditional serological biomarkers, such as autoantibodies and complement level, cannot precisely predict flares of LN [30]. Basophils, as the least abundant granulocytes, comprise <1% of circulating peripheral leukocytes [31]. Basophils play vital roles in allergic inflammation and immunity against parasitic infections by expressing the high-affinity IgE receptor FcεRI [32]. Additionally, basophils are an important source of IL-4, a type 2 cytokine, which promotes antibody production by B cells [32]. One study indicated that basophils contribute to LN development by promoting autoantibody production and that LN-like disease was suppressed when basophils were depleted in a mouse model [33]. A previous study of patients with biopsy-proven LN showed that basophil counts were decreased in patients with nephritis compared with those in patients without nephritis and that basophil counts were negatively correlated with pathological activity [34]. Based on previous and the current results, basophil counts or proportions may be a biomarker of LN.

One limitation of this study was the small sample size. Thus large prospective studies are needed to confirm and validate our findings.

## Conclusion & future perspective

In summary, we performed a feasible 14-color immunophenotyping of 1 ml peripheral blood from patients with SLE and healthy controls by flow cytometry. We found that the proportions of CD4^−^CD8^−^ T cells, NK cells and ILCs were decreased in patients with SLE and that basophil proportions were decreased in patients with LN compared with proportions in patients without LN. This 14-color immunophenotyping panel can be broadly used in the clinic to rapidly determine and monitor the immune cellular compositions of autoimmune diseases. In the future we plan to investigate the frequency of immune cell types and the absolute number of immune cells, including neutrophil subsets/eosinophils, and the absolute number of basophils, and determine the possible correlation with SLEDAI-2000.

**Summary points**

**Background**

- Systemic lupus erythematosus is a systemic autoimmune disease with various immune cellular alterations, resulting in heterogeneous clinical features.
- Detecting and monitoring immune cell alterations via a workable immunophenotyping panel is needed in clinical practice.

**Materials & methods**

- A cross-sectional, case–control study with sex- and age-matched patients and healthy controls was conducted.
- A 14-color flow cytometry panel was established to detect proportions of circulating immune mononuclear cells.
- Comparison and correlations between groups and between cellular proportions and other parameters were investigated.

**Results & discussion**

- The proportions of CD4^−^CD8^−^ T cells, natural killer cells and innate lymphoid cells were significantly decreased in patients with systemic lupus erythematosus compared with those in healthy controls.
- A previous study of patients with biopsy-proven lupus nephritis showed that basophil counts were decreased in patients with nephritis compared with those in patients without nephritis and that basophil counts were negatively correlated with pathological activity.
- In the present study, basophil proportions were significantly lower in patients with nephritis than in patients without nephritis.

**Conclusion**

- Basophil proportions or counts may be a potential biomarker of lupus nephritis.
- This 14-color immunophenotyping panel can be broadly used in clinical practice to determine and monitor immune cellular compositions in patients with autoimmune disease.
