# Supplementary material for: Basophils as a potential marker of lupus nephritis by flow cytometry
Source: Future Sci OA. 2021 Feb 16;7(5):FSO690. doi: 10.2144/fsoa-2020-0212 (PMC8147755; doi:10.2144/fsoa-2020-0212)

Supplementary files

**Supplementary Table 1.** Optimized combination of twelve commercially-available fluorochrome-conjugated antibodies to reliably quantify 14 subpopulations of immune cells.

| **Conjugation** | **Antibody** | **Clone (Company)** | **Titer** |
| --- | --- | --- | --- |
| FITC | Anti-human CD45 antibody | HI30 (BioLegend) | 1:50 |
| Percp-cy5.5 | Anti-human CD14 antibody | M5E2 (BD) | 1:50 |
| BV510 | Anti- human CD3 antibody | UCHT1 (BD) | 1:50 |
| Qdot655 | Anti-human CD4 antibody | S3.5 (Invitrogen) | 1:200 |
| PE-CY7 | Anti-human CD8 antibody | RPA-T8 (BD) | 1:100 |
| APC | Anti-human CD25 antibody | BC96 (BioLegend) | 1:20 |
| BUV395 | Anti-human CD16 antibody | 3G8 (BD) | 1:100 |
| BV605 | Anti-human CD56 antibody | NCAM16.2 | 1:100 |
| APC-CY7 | Anti-human HLA-DR antibody | L243 (BioLegend) | 1:100 |
| BV421 | Anti-human CD19 antibody | HIB19 (BD) | 1:100 |
| PE | Anti-human CD127 antibody | A019D5 (BioLegend) | 1:25 |
| BV710 | Anti-human CD123 antibody | 9F5 (BD) | 1:50 |
| Alexa Fluor® 700 | Anti-human CD11c antibody | Bu15 (BioLegend) | 1:50 |
| DAPI | Live/dead |  | 1:50 |

**Supplementary Table 2.** Definition of peripheral immune cells derived from 14-color flow cytometry panel.

| **Population name** | **Gating strategy** |
| --- | --- |
| Monocytes | CD45^+^CD3^-^CD19^-^CD14^+^ |
| Classical monocytes | CD45^+^CD3^-^CD19^-^CD14^+^ CD16^-^ |
| Intermediate monocytes | CD45^+^CD3^-^CD19^-^CD14^+^ CD16^low^ |
| Nonclassical monocytes | CD45^+^CD3^-^CD19^-^CD14^+^ CD16^hi^ |
| MyDCs | CD45^+^CD3^-^CD19^-^CD14^-^CD16^-^CD56^-^HLA-DR^+^CD11c^+^ |
| pDCs | CD45^+^CD3^-^CD19^-^CD14^-^CD16^-^CD56^-^HLA-DR^+^CD123^+^ |
| Basophils | CD45^+^CD3^-^CD19^-^CD14^-^CD16^-^CD56^-^HLA-DR^-^CD123^+^ |
| NK cells | CD45^+^CD3^-^CD19^-^CD14^-^CD56^+^ |
| CD56^dim^ NK cells | CD45^+^CD3^-^CD19^-^CD14^-^CD56^dim^ |
| CD56^bright^ NK cells | CD45^+^CD3^-^CD19^-^CD14^-^CD56^bright^ |
| T cells | CD45^+^CD3^+^CD19^-^ |
| CD4^+^ T cells | CD45^+^CD3^+^CD19^-^CD4^+^CD8^-^ |
| Tregs | CD45^+^CD3^+^CD19^-^CD4^+^CD25^hi^CD127^low^ |
| CD8^+^ T cells | CD45^+^CD3^+^CD19^-^CD4^-^CD8^+^ |
| CD4^+^CD8^+^ T cells | CD45^+^CD3^+^CD19^-^CD4^+^CD8^+^ |
| CD4^-^CD8^-^ T cells | CD45^+^CD3^+^CD19^-^CD4^-^CD8^-^ |
| B cells | CD45^+^CD3^-^CD19^+^ |
| ILCs | CD45^+^CD3^-^CD19^-^CD14^-^CD16^-^CD56^-^HLA-DR^-^CD127^+^ |

NK: natural killer; MyDCs: myeloid dendritic cells; pDCs: plasmacytoid dendritic cells; ILCs: innate lymphoid cells.

**Supplementary Table 3.** Demographic, clinical and serological features of patients with active and inactive systemic lupus erythematosus.

| **Characteristic** | **Active SLE patients**  **(n = 32)** | **Inactive SLE patients**  **(n = 28)** | **p-value** |
| --- | --- | --- | --- |
| **Age (years)** | 31.44 ± 11.05 | 39.64 ± 12.19 | 0.01 |
| **Sex, n (% female)** | 24 (85.71) | 25 (89.29) | 0.19 |
| **Disease duration (years)** | 4.07 ± 4.74 | 7.33 ± 5.65 | 0.07 |
| **Current concomitant medications** | 31 (96.88) | 27 (96.43) | 1 |
| Hydroxychloroquine, n (%) | 23 (71.88) | 13 (46.43) | 0.07 |
| Prednisone, n (%) | 31 (96.88) | 27 (96.43) | 1 |
| Current dose of prednisone, mg/d | 27.1 ± 4.79 | 33.93 ± 24.09 | 0.94 |
| Cyclophosphamide, n (%) | 8 (25) | 12 (42.86) | 0.18 |
| Cumulate dose of Cyclophosphamide, g | 1.4 ± 0.49 | 1.93 ± 0.94 | 0.85 |
| Mycophenolate Mofetil | 3 (9.38) | 0 | NS |
| Leflunomide | 0 | 1 | NS |
| **SLEDAI-2000** | 9.53 ± 3.25 | 2.39 ± 1.77 | < 0.001 |
| **System involved (% positive)** |  |  |  |
| Mucocutaneous involvements | 15 (46.88) | 11 (39.29) | 0.61 |
| Arthritis | 10 (31.25) | 11 (39.29) | 0.59 |
| Nephritis | 23 (71.88) | 15 (53.57) | 0.18 |
| Hematologic involvements | 10 (31.25) | 15 (53.57) | 0.12 |
| **Laboratory features** |  |  |  |
| ANA | 32 (100) | 28 (100) | 1 |
| ANA titer < 1:1000 | 12 (37.5) | 15 (53.57) | 0.30 |
| ANA titer ≥ 1:1000 | 20 (62.5) | 13 (46.43) | 0.30 |
| Anti-dsDNA antibody | 22 (68.75) | 12 (42.86) | 0.07 |
| Anti-dsDNA titer < 1:100 | 11 (34.38) | 10 (35.71) | 1 |
| Anti-dsDNA titer ≥ 1:100 | 11 (34.38) | 2 (7.14) | 0.01 |
| Anti-SSA antibody (%) | 17 (53.13) | 18 (64.29) | 0.44 |
| Anti-SSB antibody (%) | 14 (43.75) | 7 (25) | 0.18 |
| Anti-Sm antibody (%) | 18 (56.25) | 11 (39.29) | 0.21 |
| Anti-RNP antibody (%) | 20 (62.5) | 15 (53.57) | 0.6 |
| C3 (g/L) | 0.54 ± 0.22 | 0.53 ± 0.21 | 0.855 |
| C4 (g/L) | 0.12 ± 0.08 | 0.10 ± 0.06 | 0.374 |
| IgG (g/L) | 15.63 ± 11.26 | 14.65 ± 6.89 | 0.693 |
| IgA (mg/L) | 2257.94 ± 1144.82 | 2984.68 ± 1651.59 | 0.057 |
| IgM (mg/L) | 1112.31 ± 884.81 | 1160.89 ± 1014.29 | 0.844 |
| IgE (IU/mL) | 56.30 ± 80.11 | 38.97 ± 47.24 | 0.305 |
| ESR (mm/h) | 37.13 ± 20.31 | 29.96 ± 21.04 | 0.186 |
| CRP (mg/dL) | 11.7 ± 20.01 | 5.20 ± 6.22 | 0.089 |
| Leukocyte count (10^9^/L) | 7.16 ± 2.50 | 6.97 ± 3.30 | 0.781 |
| Lymphocyte count (10^9^/L) | 0.99 ± 0.45 | 1.31 ± 1.56 | 0.272 |
| Granulocyte count(10^9^/L) | 5.75 ± 2.48 | 5.01 ± 3.25 | 0.273 |

Data are expressed as absolute numbers (%) or the means ± standard deviation. SLE: systemic lupus erythematosus; SLEDAI-2000: SLE disease activity index 2000; HC: healthy controls; ANA: anti-nuclear antibody. dsDNA: double-strand DNA; SSA: Sjögren’s-syndrome-related antigen A; SSB: Sjögren’s-syndrome-related antigen B; Sm: Smith; RNP: ribonucleoprotein; C3/4: complement component 3/4; CRP: C-reactive protein; Ig: immunoglobulin; ESR: erythrocyte sedimentation rate.

**Supplementary Table 3.** Demographic, clinical and serological features of patients with and without lupus nephritis (LN).

| **Characteristic** | **Patient with LN**  **(n = 38)** | **Patient without LN**  **(n = 22)** | **p-value** |
| --- | --- | --- | --- |
| **Age (years)** | 35.94 ± 12.54 | 38.83 ± 11.63 | 0.31 |
| **Sex, n (% female)** | 31 (81.58) | 18 (81.82) | 0.762 |
| **Disease duration (years)** | 6.25 ± 1.31 | 3.59 ± 1.18 | 0.213 |
| **Current concomitant medications** | 38 (1) | 20 (90.91) | 0.131 |
| Hydroxychloroquine, n (%) | 21 (55.26) | 15 (68.18) | 0.416 |
| Prednisone, n (%) | 38 (1) | 20 (90.91) | 0.131 |
| Current dose of prednisone, mg/d | 36.86 ± 25.49 | 44.21 ± 17.74 | 0.195 |
| Cyclophosphamide, n (%) | 10 (26.32) | 10 (45.45) | 0.161 |
| Cumulate dose of Cyclophosphamide, g | 2.68 ± 2.99 | 1.60 ± 0.84 | 0.946 |
| Mycophenolate Mofetil | 3 (7.89) | 0 | 0.292 |
| Leflunomide | 1 (2.63) | 0 | 1 |
| **SLEDAI-2000** | 6.34 ± 4.50 | 4.95 ± 3.95 | 0.14 |
| **System involved (% positive)** |  |  |  |
| Mucocutaneous involvements | 18 (46.88) | 8 (36.36) | 0.433 |
| Arthritis | 14 (31.25) | 7 (31.82) | 0.783 |
| Hematologic involvements | 13 (31.25) | 12 (54.55) | 0.175 |
|  |  |  |  |
| **Laboratory features** |  |  |  |
| ANA | 38 (100) | 22 (100) | NS |
| ANA titer < 1:1000 | 19 (50) | 8 (36.36) | 0.42 |
| ANA titer ≥ 1:1000 | 19 (50) | 14 (63.64) | 0.42 |
| Anti-dsDNA antibody | 10 (26.32) | 11 (50) | 0.093 |
| Anti-dsDNA titer < 1:100 | 7 (18.42) | 8 (36.36) | 0.137 |
| Anti-dsDNA titer ≥ 1:100 | 31 (81.58) | 14 (63.64) | 0.137 |
| Anti-SSA antibody (%) | 23 (60.53) | 12 (54.55) | 0.787 |
| Anti-SSB antibody (%) | 16 (42.11) | 5 (22.73) | 0.166 |
| Anti-Sm antibody (%) | 21 (55.26) | 8 (36.36) | 0.188 |
| Anti-RNP antibody (%) | 27 (71.05) | 8 (36.36) | 0.014 |
| C3 (g/L) | 0.56 ± 0.19 | 0.50 ± 0.25 | 0.234 |
| C4 (g/L) | 0.12 ± 0.08 | 0.09 ± 0.06 | 0.09 |
| IgG (g/L) | 13.32 ± 1.42 | 16.32 ± 1.97 | 0.215 |
| IgA (mg/L) | 2882.78 ± 314.03 | 2701.29 ± 342.29 | 0.7 |
| IgM (mg/L) | 1280.33 ± 229.75 | 1230.86 ± 257.93 | 0.837 |
| IgE (IU/mL) | 250.34 ± 229.75 | 129.07 ± 73.11 | 0.301 |
| ESR (mm/h) | 31.26 ± 20.13 | 38.14 ± 21.67 | 0.140 |
| CRP (mg/dL) | 9.67 ± 18.01 | 6.93 ± 9.70 | 0.384 |
| Leukocyte count (10^9^/L) | 7.28 ± 2.96 | 6.71 ± 2.80 | 0.369 |
| Lymphocyte count (10^9^/L) | 1.21 ± 1.39 | 1.03 ± 0.42 | 0.400 |
| Granulocyte count(10^9^/L) | 5.47 ± 3.01 | 5.25 ± 2.71 | 0.726 |

Data are expressed as absolute numbers (%) or the means ± standard deviation. SLE: systemic lupus erythematosus; SLEDAI-2000: SLE disease activity index 2000; HC: healthy controls; ANA: anti-nuclear antibody. dsDNA: double-strand DNA; SSA: Sjögren’s-syndrome-related antigen A; SSB: Sjögren’s-syndrome-related antigen B; Sm: Smith; RNP: ribonucleoprotein; C3/4: complement component 3/4; CRP: C-reactive protein; Ig: immunoglobulin; ESR: erythrocyte sedimentation rate.

**Supplementary Figure 1.** The color compensation matrix graph of 14-color flow cytometry panel.


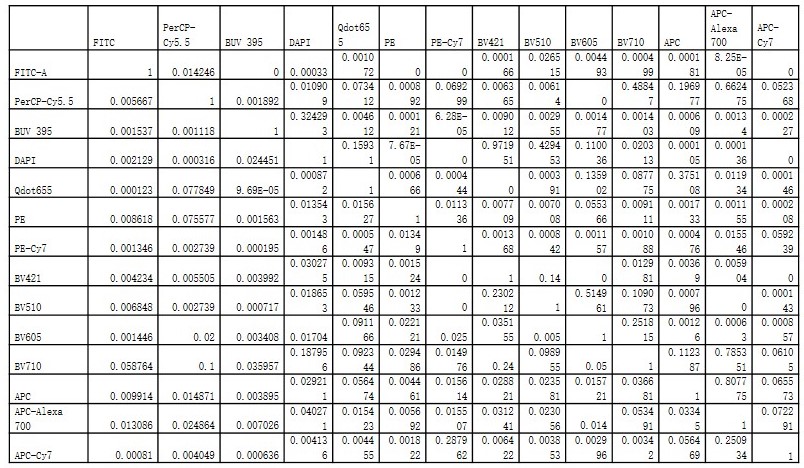

Supplement: Supplementary file 1 [file fsoa-07-690-s1.doc]
